# Association between Resistin Gene Polymorphisms and Atopic Dermatitis

**DOI:** 10.3390/biom8020017

**Published:** 2018-03-27

**Authors:** Saleem A. Banihani, Khawla F. Abu-Alia, Omar F. Khabour, Karem H. Alzoubi

**Affiliations:** 1 Department of Medical Laboratory Sciences, Jordan University of Science and Technology, Irbid 22110, Jordan; khawlaabualia@gmail.com (K.F.A.-A.); khabour@just.edu.jo (O.F.K); 2 Department of Clinical Pharmacy, Jordan University of Science and Technology, Irbid 22110, Jordan; khalzoubi@just.edu.jo

**Keywords:** resistin gene, polymorphisms, atopic dermatitis

## Abstract

Atopic dermatitis (AD) is a chronic, relapsing, and inflammatory skin disorder. It is characterized by an inappropriate skin barrier function, allergen sensitization, and recurrent skin infections. Resistin is an adipokine expressed mainly in macrophages and monocytes; it has a role in the inflammatory process and is associated with multiple inflammatory human diseases; however, only few studies linked resistin to atopic dermatitis. This study tested the association between G>A (rs3745367) and C>T (rs3219177) single nucleotide polymorphisms (SNPs) of the *RETN* gene with atopic dermatitis. In addition, it explored the relationship between serum resistin protein and atopic dermatitis. To achieve objectives of this study, 162 atopic dermatitis patients and 161 healthy participants were recruited in the study. A significant association was detected between rs3745367 and atopic dermatitis with age and gender specificity (*p* < 0.05), while no significant association between rs3219177 and atopic dermatitis was found (*p* > 0.05). For the serum resistin levels, a significant decrease was indicated in atopic dermatitis patients compared to healthy subjects (*p* < 0.05). In conclusion, rs3745367 may play a gender and age-specific role in atopic dermatitis. In addition, the significant decrease in the resistin protein level confirmed this association.

## 1. Introduction

Atopic dermatitis (AD), also called eczema, is a chronic inflammatory skin disease characterized by an inappropriate skin barrier function, recurrent skin infections, and allergen sensitization [1]. Though AD affects patients of all ages, it is mainly prevalent among children. The prevalence of AD, in general, has increased threefold since the 1960s. It is estimated that approximately 10–20% of children in industrialized countries are affected by AD [2,3]. In addition, developing countries also show increased prevalence rates of AD [4]. The exact reason for the increase in the prevalence of AD is not well identified, but many reports showed that both environmental and genetic factors play a role in the expression of this disease [5]. Other factors such as level of education, small family size, high income, increased antibiotic intake, and migration from rural to urban areas might also be associated with AD [6].

Adipocytokines are cell signaling substances secreted by adipose tissues [7]. They include hormones (i.e., adiponectin and leptin), cytokines such as tumor necrosis factor-alpha (TNF-𝛼), interleukin-6 (IL-6), interleukin-10 (IL-10), and visfatin. Adipocytokines also include proteins like apelin and resistin, which contribute to many physiological and pathological processes [8].

Resistin (encoded by *RETN*) is a cysteine-rich peptide hormone, also named adipose tissue-specific secretory factor. It belongs to a small gene family of resistin-like molecules [9]. It was discovered in mice 16 years ago and called “adipose tissue-specific secretory factor” [10], but the name “resistin” is used due to its association with insulin resistance [11]. Human *RETN* is expressed in monocytes and macrophages rather than in adipose tissues in animals [12]. It has been shown that the levels of plasma resistin are interrelated to inflammation, but not the amount of adipose tissues [13]. Several studies suggest that resistin is associated with multiple inflammatory human diseases including diabetes and cardiovascular diseases [14,15]. However, to date, only a few studies linked resistin to atopic dermatitis [16].

Several studies have revealed pro-inflammatory properties of resistin. For example, in humans, resistin was found to boost the expression of IL-6 and TNF-𝛼 by mononuclear cells [7,13]. Additionally, resistin was found to promote the expression of the pro-inflammatory adhesion molecules such as intercellular adhesion molecule 1, vascular cell adhesion molecule 1, and pentraxin 3 in vascular endothelial cells, thereby promoting the adhesion of leukocytes [7].

One of the hypotheses that suggests the pathophysiology of AD disease is the immunological hypothesis, and it argues that the allergic state results in increased production of proinflammatory cytokines TNF-α, IL-1, and IL-6, which affects the expression of *RETN* gene in human peripheral blood mononuclear cells [17,18]. Thus, we hypothesized that patients with AD may have abnormal levels of serum resistin and there might be an association between *RETN* gene polymorphisms and the development of AD. Therefore, the aim of this study was to compare serum resistin levels among AD patients to that of healthy controls, then, to examine possible association between *RETN*: +299 G>A (rs3745367) and +157 C>T (rs3219177) single nucleotide polymorphisms (SNPs) and AD among Jordanians. Previous literature has shown an association between rs3745367 and other polymorphisms in the *RETN* gene with acne vulgaris development [19,20]. Current results might enhance the knowledge related to the contribution of resistin to the skin diseases.

## 2. Materials and Methods 

### 2.1. Subjects

One hundred and sixty-two patients with AD were recruited in this case-control study. The control group consisted of one hundred and sixty-one age and sex matched healthy subjects. Exclusion criteria for the patients’ group were the presence of skin diseases other than AD and obesity (defined as body mass index > 29.9). Actually, obese patients were excluded as they have higher levels of resistin compared to non-obese [21]. In addition, a positive association has been reported between obesity and AD [22,23].

The age of the participants was between newborn and 60 years-old. All subjects were recruited from King Abdulla University Hospital, the Health Center at Jordan University of Science and Technology (JUST), and the Ministry of Health Hospitals in the North of Jordan. All subjects provided written informed consent under a protocol approved by the JUST Institutional Review Board (ethical code: IRB19-80-2014). A structured questionnaire interview was conducted to collect data on the characteristics of the study participants. Other related data regarding clinical characteristics of recruited patients were obtained from the medical records. Such characteristics included height, weight, use of medications, presence of other diseases, and smoking status.

### 2.2. Sample Collection and Handling

Two blood specimens were obtained from each subject. Five milliliters of whole blood were collected in an ethylenediaminetetraacetic acid (EDTA) tube for the molecular analysis, and another five milliliters were collected in a plane tube for the biochemical analysis. The storage conditions for EDTA samples were −20 °C, and for serum specimens obtained from plane tubes, −80 °C.

### 2.3. Genetic Analysis

#### DNA Extraction

DNA was isolated from collected blood samples using Wizard® Genomic DNA Purification Kit (Cat# A1125 Madison, WI, USA) according to the manufacturer’s instructions.

### 2.4. Molecular Analysis 

Two SNPs were analyzed, SNP +299 G>A (rs3745367) and SNP +157 C>T (rs3219177) using polymerase chain reaction (PCR), followed by restriction fragment length polymorphism (RFLP).

#### 2.4.1. PCR Procedure

Amplification of target sequences was done using PCR, which was performed in a total volume of 25 μL: 12.5 μL of commercial master mix containing Taq DNA polymerase, MgCl_2_, dNTPs, and buffer (Promega, Madison, WI, USA), 1 μM of each forward and reverse primer, and 5 ng of DNA sample. Primers’ sequences for each SNP, and the PCR conditions are listed in Table 1. Five microliters from each PCR sample was loaded on 2% agarose gel. Electrophoresis was carried out at 140 volts for 60 min. PCR amplified fragments were then detected by UV light and ethidium bromide.

#### 2.4.2. Restriction Fragment Length Polymorphism

To genotype *RETN* SNPs, a restriction fragment length polymorphism (RFLP) technique was used. About 2 μL nuclease free water, 2 μL Cut Smart buffer, and 0.5 μL of restriction enzyme were mixed with 10 μL PCR amplified samples, and then incubated at 37 °C overnight. Finally, the samples were loaded on 3% agarose gel and electrophoresed at 140 volts for 80 min. RFLP restriction enzymes and sizes for each SNP restricted fragments are listed in Table 1.

### 2.5. Resistin Serum Level

Patient (*n* = 75) and control (*n* = 75) serum samples were analyzed for resistin concentrations using R&D Systems ELISA kit (DuoSet^®^ Human Resistin Immunoassay, Minneapolis, MN, USA) as per the kit manufacturer’s instructions. In brief, serum was diluted by eightfold and then 100 μL of the diluted samples were used in the assay. The absorbance (at 450 nm) was measured using ELx800 plate reader (BioTek Instruments, Inc., Winooski, VT, USA). Concentrations of resistin in the samples were deducted from the standard curve that was constructed based on known concentrations of resistin samples provided by the kit. 

### 2.6. Statistical Analysis 

The statistical software SPSS (version 23, Manufacturer, Chicago, IL, USA) was used to examine the distribution of alleles, genotypes and agreement with Hardy–Weinberg equilibrium of examined SNPs. This was performed using chi-square test/Fisher Exact test. Resistin protein levels in the serum were expressed as the mean ± standard error of the mean (SEM) and were analyzed using Student's *t*-test. *p* < 0.05 was considered statistically significant. 

## 3. Results

### 3.1. Characteristics of the Participants

Control and patient’s groups were matched for age and gender (Table 2).

### 3.2. Association between Resistin Gene Polymorphisms and Atopic Dermatitis

Table 3 shows the frequencies of genotypes and alleles for rs3219177 and rs3745367 resistin gene SNPs in patients and control groups. The rs3219177 SNP showed no difference in genotypes frequency (*p* = 0.219) and alleles frequency (*p* = 0.625) between the two groups. In contrast, genotypes for rs3745367 SNP revealed a significant difference (*p* = 0.023), whereas allele frequency showed marginal differences between the two groups (*p* = 0.091). Thus, the rs3745367 SNP appears to be associated with AD.

It has been shown that gender is a variable that affects the genotype of *RETN* gene [24]. Therefore, the association between *RETN* gene SNPs and AD was retested according to gender.

Genotype and allele frequencies for rs3745367 SNP of *RETN* gene were significantly associated with AD in males (genotype frequency *p* = 0.007, allele frequency *p* = 0.0003, Table 4), but not in females (genotype frequency *p* = 0.82, allele frequency *p* = 0.824, Table 5). However, no differences were found between AD and the control group when considering the gender in rs3219177 SNP of *RETN* gene in males (genotype frequency *p* = 0.255, allele frequency *p* = 0.755, Table 4), as well as in females (genotype frequency *p* = 0.731, allele frequency *p* = 0.708, Table 5).

To investigate whether the association between *RETN* SNPs and AD is affected by age groups, genotypes and allele distributions were examined in each age group separately. Genotype for rs3745367 SNP of *RETN* gene showed a significant difference between AD and control groups in newborn and ten-year-old children (*p* = 0.028) and a marginal difference in allele frequency (*p* = 0.098). No significant difference was found in the age group between eleven and twenty years old (genotype frequency *p* = 0.243, allele frequency *p* = 0.39), as well as in the age group of more than twenty years old (genotype frequency *p* = 0.516, allele frequency *p* = 0.918) as shown in Table 6. With respect to rs3219177 SNP of *RETN* gene, genotypes and alleles did not show significant differences in any of the age groups between patients and controls (genotype frequencies; *p* = 0.24 for new born to 10 years, *p* = 0.798 between eleven and twenty years, and *p* = 1.00 for the age group of more than twenty years; allele frequencies: *p* = 0.667 for new born to 10 years, *p* = 0.598 between eleven and twenty years, and *p* = 0.83 for the age group of more than twenty years old, Table 6). The distribution of all examined SNPs in this study was in accordance with Hardy–Weinberg equations.

### 3.3. Association between Resistin Protein Level and AD

**Figure 1:**
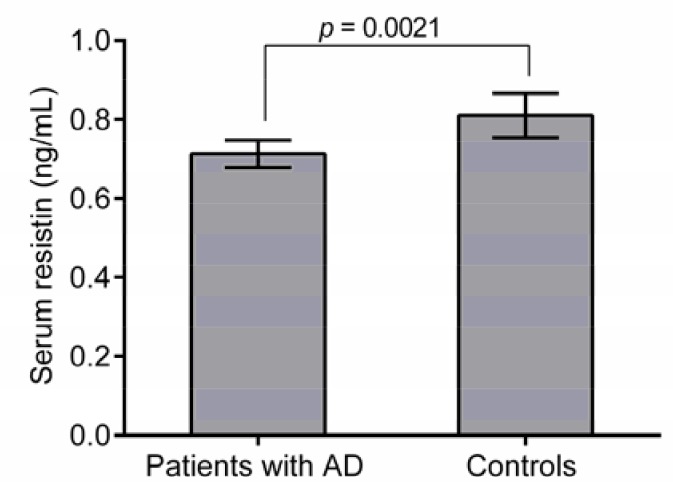
Serum resistin levels in AD and control groups. The data are from 150 subjects (75 patients and 75 controls). Results are mean + SEM. A significant decrease in serum resistin levels in AD patients was detected compared to the control groups (*p* = 0.0002).

To examine the association between resistin and AD, serum resistin concentrations were determined using ELISA. The mean serum resistin level was 6.1 ± 0.36 ng mL^-1^ in patients and 8.1 ± 0. 56 ng mL^-1^ (*p* = 0.0002; Figure 1) for the control group. Thus, AD seems to be associated with significant decrease in resistin serum levels. Further analysis showed that resistin levels are significantly lower in patients with GG genotype of rs3745367 compared to the control group (*p* = 0.021). However, no significant differences were found in resistin levels of other genotypes between two groups (*p* > 0.05).

## 4. Discussion

Several studies suggested that resistin is associated with multiple human diseases such as diabetes [25] and cardiovascular diseases (e.g., arteriosclerosis and heart failure) [26,27,28], but only few studies linked it to AD [16]. Actually, high serum resistin concentrations are significantly correlated with adiposity and low insulin sensitivity in obese people [29]. In addition, high serum resistin was found to be a risk factor for cardiovascular disease in patients with type 2 diabetes [30]. Latterly, resistin has received a great deal of attention because of its role in insulin resistance and type 2 diabetes [31]. Resistin plays an important role in inflammatory process and is naturally expressed at different levels in different tissues [12,32,33].

Results from this study showed a significant association between rs3745367 SNP in the *RETN* gene and AD. In contrast, no association between rs3219177 SNP and AD was found. The concentration of serum resistin was strongly associated with AD. Resistin concentrations were lower in patients with AD compared to healthy individuals.

Resistin was found to be expressed in basal sebocytes where it plays a role in inflammatory skin conditions [34]. A previous study showed that rs1862513 SNP in the *RETN* gene was strongly associated with familial acne vulgaris in patients from Pakistan [20]. The clinical significance of other SNPs is extended in the current study, which indicated the association between rs3745367 SNP in the *RETN* gene and AD. In the current study, results showed that rs3745367 SNP in the *RETN* gene is significantly associated with male AD patients, but not with females. Similarly, a previous study showed that levels of serum resistin were different in male versus female AD patients. A previous study from our laboratory showed an association between male longevity and SNPs in adipocytokines other than resistin [35]. Thus, a possible relationship between diseases and resistin according to gender could exist. This could be related to the difference in amount and functionality of adipose tissues in each gender [36]. In addition, variations in hormonal signaling pathways and immune response between both genders could account for such relationship [24,37]. Furthermore, age differences were found in the association between rs3745367 SNP and AD. Results showed only the age group between newborn children and 10 years old is associated with rs3745367 SNP in the *RETN* gene. 

Current results did not show of any association in rs3219177 SNP of *RETN* gene between AD and healthy controls. Thus, the clinical significance of rs3219177 SNP of *RETN* gene in AD seems to be limited. In accordance, a study from Finland showed no association between rs3219177 SNP of *RETN* gene and resistin levels in patients with hypertension [38]. 

In the present investigation, AD was associated with a significant decrease in serum concentrations of resistin protein. Positive association was reported in study from South Korea that indicated lower resistin levels in patients with atopic asthma compared to control groups [39]. In contrast, a couple of studies on Turkish patients with systemic sclerosis [40], and Iranian patients with psoriasis [41], showed higher resistin levels in patients compared to control groups. Thus, alterations in the serum resistin levels might predispose to different sets of diseases. It is worth mentioning that, in the current study, resistin levels are significantly lower in patients with GG genotype of rs3745367 compared to control groups. Further studies are required to investigate how this genotype differentially affects resistin levels in the patient group.

Furthermore, increases in resistin levels were associated with other diseases such as cystic fibrosis [42], vascular diseases [43], and metabolic disorders [44]. Thus, the association between resistin levels and various diseases might display a bell shape model where both increases and decreases in a specific protein can be associated with different sets of diseases. This phenomenon was observed for the related adiponectin protein levels [45]. Alternatively, factors that might contribute to the development of atopic dermatitis might negatively influence resistin levels. For example, exercise has been shown to trigger or worsen atopic dermatitis due to sweating [46,47], and it was reported to decrease levels of resistin [48,49]. Another example is impairment of thyroid function, which was shown to decrease resistin [50], and to augment atopic dermatitis [51]. Thus, the exact mechanisms by which decreases in resistin levels contribute to the etiology of atopic dermatitis need further investigations. For example, resistin increases transcriptional events that augment the expression of several pro-inflammatory cytokines, including IL-1, IL-6, IL-12, and TNF-α [52,53]. Resistin also upregulates intercellular adhesion molecule-1 and chemokine (C-C motif) ligand 2, which participate in leukocytes’ recruitment pathways at infection sites [54]. Upregulation of resistin has been shown to be mediated by interleukins and microbial antigens like lipopolysaccharide, which can be identified by leukocytes [55]. Thus, downregulation of resistin might be associated with the immune response and the subsequent development of AD. Further studies are required to understand resistin’s role in the pathogenesis of AD. 

Among the limitations of the current investigation is that only two polymorphisms (rs3745367 and rs3219177) were investigated. Extending the present study in future investigation to include other polymorphisms in the *RETN* gene such as rs1862513 that has been shown to be associated with acne is strongly recommended [19,20]. 

## 5. Conclusions

In conclusion, rs3745367 *RETN* SNP may play a role in the development of AD in a gender and age specific manner. In addition, the current finding that resistin’s circulatory levels were modulated during AD strengthens this after-mentioned genetic association.

## Figures and Tables

**Table 1:** Single nucleotide polymorphisms (SNPs ID), PCR primers, PCR conditions and restriction conditions.

SNP ID	Primer Sequence (5’–3’)	PCR Annealing T (°C)	Restriction Enzyme, Incubation Conditions	Fragment Length (bp)
rs3745367	F: GGAAGAAGCCATCAATGAGAGG R: CCTGTTGGTTTGGAGCTAGGTC	58	Alu I, 37 °C overnight	GG➔ 14, 71, 243 GA➔ 14, 71, 85, 158, 243 AA➔ 14, 71, 85, 158
rs3219177	F: AGTGACAGCTGCTCCTGCG R: ATGAGATTTGGTGAGCGCT	58	BspCN I, 37 °C overnight	CC➔ 48, 376, 351 CT➔ 48, 54, 322, 376, 424 TT➔ 48, 54, 322, 376

**Table 2:** General characteristics of the participants.

Variable	Control *n* (%)	Patient *n* (%)	*p*-Value
Gender			
Male	82 (50.9%)	82 (50.6%)	0.955
Female	79 (49.1%)	80 (49.4%)	
Total	161	162	
Age groups			
New Born–10 years	94 (58.4%)	92 (56.8%)	0.885
11–20 years	36 (22.4%)	40 (24.7%)	
>20	31 (19.2%)	30 (18.5%)	

**Table 3:** Genotype and allele frequencies of rs3219177 and rs3745367 of *RETN* gene in atopic dermatitis (AD) subjects and controls.

Genotypes and Alleles	Control *n* (%)	Patient *n* (%)	*p*-Value
rs3219177			
CC	113 (70.2%)	107 (66.0%)	0.219 NS
TT	4 (2.5%)	1 (0.6%)	
CT	44 (27.3%)	54 (33.4%)	
Allele C	157 (76.6%)	161 (74.5%)	0.625 NS
Allele T	48 (23.4%)	55 (25.5%)	
rs3745367			
GG	52 (32.3%)	76 (46.9%)	0.023
AA	32 (19.9%)	22 (13.6%)	
GA	77 (47.8%)	64 (39.5%)	
Allele G	129 (54.2%)	140 (61.9%)	0.091
Allele A	109 (45.8%)	86 (38.1%)	

NS: not significant.

**Table 4:** Frequencies of *RETN* genotypes and alleles in males.

Genotypes and Alleles	Control *n* (%)	Patient *n* (%)	*p*-Value
rs3219177			
CC	57 (69.5%)	54 (65.1%)	0.255
TT	4 (4.9%)	1 (1.2%)	
CT	21 (25.6%)	28 (33.7%)	
Allele C	78 (75.7%)	82 (73.9%)	0.755
Allele T	25 (24.3%)	29 (26.1%)	
rs3745367			
GG	26 (31.7%)	44 (53%)	0.007
AA	14 (17.1%)	5 (6%)	
GA	42 (51.2%)	34 (41%)	
Allele G	40 (54.8%)	78 (66.7%)	0.0003
Allele A	56 (45.2%)	39 (33.3%)	

**Table 5:** Frequencies of *RETN* genotypes and alleles in females.

Genotypes and Alleles	Control *n* (%)	Patient *n* (%)	*p*-Value
rs3219177			
CC	56 (70.9%)	53 (67.1%)	0.731
TT	0 (0%)	0 (0%)	
CT	23 (29.1%)	26 (32.9%)	
Allele C	79 (77.5%)	79 (75.2%)	0.708
Allele T	23 (22.5%)	26 (24.8%)	
rs3745367			
GG	27 (34.2%)	30 (38%)	0.82
AA	17 (21.5%)	17 (21.5%)	
GA	35 (44.3%)	32 (40.5%)	
Allele G	62 (54.4%)	62 (55.9%)	0.824
Allele A	52 (45.6%)	49 (44.1%)	

**Table 6:** The frequency of *RETN* genotypes and alleles among different age groups.

Age Groups						
Genotypes and Alleles	New Born–10 years		11–20 years		>20 years	
**rs3219177**	Control	Patient	Control	Patient	Control	Patient
CC	64 (68.1%)	58 (63%)	27 (75%)	27 (67.5%)	22 (71%)	22(73.3%)
TT	2 (2.1%)	0 (0%)	1 (2.8%)	1 (2.5%)	1 (3.2%)	0 (0%)
CT	28 (29.8%)	34 (37%)	8 (22.2%)	12 (30%)	8 (25.8%)	8 (26.7%)
*p*-value	0.24		0.798		1.00	
Allele C	92 (75.4%)	92 (73%)	35 (79.5%)	39 (75%)	30 (77%)	30(78.9%)
Allele T	30 (24.6%)	34 (27%)	9 (20.5%)	13 (25%)	9 (23%)	8 (21.1%)
*p*-value	0.667		0.598		0.83	
**rs3745367**						
GG	30 (31.9%)	47 (51.1%)	11 (30.6%)	16 (40%)	11 (35.5%)	13(43.3%)
AA	19 (20.2%)	12 (13%)	9 (25%)	4 (10%)	4 (12.9%)	6 (20%)
GA	45 (47.9%)	33 (35.9%)	16 (44.4%)	20 (50%)	16 (51.6)	11(36.7%)
*p*-value	0.028		0.243		0.516	
Allele G	75 (54%)	80 (64%)	27 (51.9%)	36 (60%)	27 (57.4%)	24(58.5%)
Allele A	64 (46%)	45 (36%)	25 (48.1%)	24 (40%)	20 (42.6%)	17(41.5%)
*p*-value	0.098		0.39		0.918	
